# Does seasonality drive spatial patterns in demography? Variation in survival in African reed warblers *Acrocephalus baeticatus* across southern Africa does not reflect global patterns

**DOI:** 10.1002/ece3.958

**Published:** 2014-02-23

**Authors:** Dorine YM Jansen, Fitsum Abadi, Doug Harebottle, Res Altwegg

**Affiliations:** 1 Animal Demography Unit, Department of Biological Sciences, University of Cape TownRondebosch, 7701, South Africa; 2 Centre for Statistics in Ecology, Environment and Conservation, Department of Statistical Sciences, University of Cape TownRondebosch, 7701, South Africa; 3 South African National Biodiversity InstituteClaremont, 7735, South Africa

**Keywords:** Avian life history, capture–mark–recapture, JAGS, multistate state-space, seasonality, spatial variation

## Abstract

Among birds, northern temperate species generally have larger clutches, shorter development periods and lower adult survival than similarly-sized southern and tropical species. Even though this global pattern is well accepted, the driving mechanism is still not fully understood. The main theories are founded on the differing environmental seasonality of these zones (higher seasonality in the North). These patterns arise in cross-species comparisons, but we hypothesized that the same patterns should arise among populations within a species if different types of seasonality select for different life histories. Few studies have examined this. We estimated survival of an azonal habitat specialist, the African reed warbler, across the environmentally diverse African subcontinent, and related survival to latitude and to the seasonality of the different environments of their breeding habitats. Data (1998–2010) collected through a public ringing scheme were analyzed with hierarchical capture-mark-recapture models to determine resident adult survival and its spatial variance across sixteen vegetation units spread across four biomes. The models were defined as state-space multi-state models to account for transience and implemented in a Bayesian framework. We did not find a latitudinal trend in survival or a clear link between seasonality and survival. Spatial variation in survival was substantial across the sixteen sites (spatial standard deviation of the logit mean survival: 0.70, 95% credible interval (CRI): 0.33–1.27). Mean site survival ranged from 0.49 (95% CRI: 0.18–0.80) to 0.83 (95% CRI: 0.62–0.97) with an overall mean of 0.67 (95% CRI: 0.47–0.85). A hierarchical modeling approach enabled us to estimate spatial variation in survival of the African reed warbler across the African subcontinent from sparse data. Although we could not confirm the global pattern of higher survival in less seasonal environments, our findings from a poorly studied region contribute to the study of life-history strategies.

## Introduction

In the 170 years since German explorers first described substantially smaller clutch sizes in South American birds compared with those found in Europe (Skutch 1985), empirical data of a latitudinal gradient in many avian life-history traits have accumulated in both the New World (Yom-Tov et al. 1994; Young 1994; Johnston et al. 1997; Ricklefs 1997; Ghalambor and Martin 2001; Tarwater and Brawn 2010) and the Old World (Moreau 1944; Lack 1968; Rowley and Russell 1991), although few studies have compared survival between the Old World northern and southern hemisphere (Yom-Tov et al. 1992; Peach et al. 2001; Schaefer et al. 2004; Stevens et al. 2013). Southern hemisphere and tropical species, particularly passerines, are characterized by smaller clutch sizes, higher nest predation, several nesting attempts, longer development periods and parental care, and thus higher juvenile survival, and higher adult survival than closely related northern, temperate species of similar body mass (Martin 1996; also see Skutch 1985 and McNamara et al. 2008 for more references). Even though not all studies have found these patterns (Karr et al. 1990; Brawn et al. 1995; Sandercock et al. 2000; Ricklefs and Shea 2007; Blake and Loiselle 2008; Ricklefs et al. 2011), it is still the generally accepted paradigm.

Life-history theory predicts that with limited available resources each individual must balance the energy demands of growth, maintenance, and reproduction, to maximize fitness in its natural and demographic environment (Gadgil and Bossert 1970). How the four components of food supply, reproductive rate, mortality/survival, and population density interact to drive the evolution of life history, and thus explain the latitudinal gradient in life-history strategies, is the subject of lively debate to this day, since the first tentative hypothesis was suggested by Hesse in 1922 (reviews in Martin 1996; Ricklefs 2000; McNamara et al. 2008; Skutch 1985). The three main hypotheses centre on the seasonality of the environment, which shows a latitudinal gradient in day length and climatic extremes with stability around the equator and ever more extremity toward the poles. Lack (1947) proposed that longer day length during the breeding season in the North would enable parents to raise larger broods, leading to the evolution of larger clutch sizes, and correspondingly lower adult survival. Skutch (1949) argued that reproduction rate was adjusted to mortality, which must be higher in the North due to the hazards of migration or winter and lower in the South because of the observed smaller clutch sizes and stable population densities. He also proposed that higher nest predation in the South would select for smaller and thus easier to conceal broods. Ashmole (1963) contended that northern temperate climates – with a highly varying food supply leading to high mortality – would decrease population density during the nonbreeding season, and thus decrease food competition during the breeding season, leading to a higher reproductive rate. Findings continue to emerge to support or dispute one or other of these hypotheses (Ricklefs 1980; Dijkstra et al. 1990; Ferretti et al. 2005; Halupka and Greeney 2009; Rose and Lyon 2013). The patterns are apparent across species, but if the hypotheses above hold, that is, types of seasonality select for particular life histories, we should also expect to see the same patterns within genera of closely related species and within species where populations inhabit areas that differ in seasonality. Finding these same patterns would confirm the generality of the paradigm.

The Old World Acrocephalidae family of reed and bush warblers is a phylogenetically homogeneous group and one of the most extensively studied avian groups and as such well suited to comparative studies of life-history strategies (Leisler and Schultze-Hagen 2011). The true reed warblers *Acrocephalus* occur sympatrically in wetlands – a global, azonal habitat that varies in extent, composition, density, and height among biomes (Leisler and Schultze-Hagen 2011; Nel and Driver 2012). Within this genus, the subgroup of six small, plain-backed marsh warblers contains the Eurasian reed warbler (*Acrocephalus scirpaceus*, Hermann) and the African reed warbler (*Acrocephalus baeticatus*, Vieillot), which are deemed sister taxa or conspecific depending on the sample, methodology, and threshold of genetic distance used to separate species (Helbig and Seibold 1999; Fregin et al. 2009, 2012). The difference in research extent between these two insectivorous warblers is striking. Most details of the breeding ecology of the African reed warbler were gathered in a 1-year study in one study area by Eising et al. (2001), and to date, survival was estimated for one population in Malawi (Peach et al. 2001). In contrast, long-term studies have covered most biological and ecological aspects of the Eurasian reed warbler's life history, resulting in findings representative of the species and not merely a “snapshot” of the observed population (Leisler and Schultze-Hagen 2011; Fitzsimmon 2013).

The African reed warbler is a tropical and southern, partial intermediate migrant (migratory roughly below 26°S) thought to migrate to Central Africa during the austral winter (June–August); the Eurasian reed warbler is a northern, temperate long-distance migrant wintering mainly in West and East Africa and as far south as northern Angola with rare sightings in South Africa (Dean 2005; Herremans 2005; Kennerley and Pearson 2010). Apart from nest predation, the comparison between these two species shows patterns consistent with the generally accepted latitudinal trend in avian life-history traits (Table 1; page numbers indicate several sources).

**Table 1:** Comparison of life-history traits of the African reed warbler (ARW) and the Eurasian reed warbler (ERW) sourced from published studies. ?, no data are available.

Trait	ARW	ERW	References
Clutch size (commonly)	2–3	4–5	1
Nest predation per breeding season	20%	28–95%	2
Nesting attempts (after brood fails)	?	1–5	3
Incubation (days)	12–14	9–12	4
Fledging (days)	12–14	10–13	5
Parental care after fledging (days)	?	10–14	6
Juvenile survival (mean probability)	?	0.22	7
Adult survival (mean probability)	0.77	0.51, 0.56, 0.59, 0.61, 0.46, 0.56	8

1 ARW – Urban et al. 1997; Eising et al. 2001; ERW – p. 211 Simms 1985;

2 ARW – Eising et al. 2001; ERW – p. 106 Honza et al. 1998; Halupka et al. 2008;

3 ERW – p. 185 Schultze-Hagen et al. 1996; Halupka et al. 2008;

4 ARW – Urban et al. 1997; Eising et al. 2001; ERW – Simms 1985; Halupka et al. 2008; Kennerley and Pearson 2010;

5 ARW – Urban et al. 1997; Eising et al. 2001; ERW – Simms 1985; Halupka et al. 2008; Kennerley and Pearson 2010;

6 ERW – Kennerley and Pearson 2010;

7 ERW – Coehoorn et al. 2011;

8 ARW – Peach et al. 2001; ERW – p. 213 Simms 1985; Buckland and Baillie 1987; Peach et al. 1990; Coehoorn et al. 2011; Kew and Leech 2013.

As it now appears that the pattern holds across these very closely related species, a more powerful test of the theory would be to compare populations of a single species occurring in environments with different seasonality. This would facilitate understanding of causal relationships, ecological constraints, population density regulation, and the evolution of life-history traits (Frederiksen et al. 2005; Dhondt 2001; examples Thaxter et al. (2006) and Saracco et al. 2012). Additionally, data collected following one protocol and curated by a single institution would yield well-founded results (Frederiksen et al. 2005). The objective of this study was to investigate spatial variation in adult survival of the African reed warbler found in wetlands across the southern African subcontinent, which is, relative to its size, one of the most environmentally diverse areas in the world (Allan et al. 1997). We used ringing data collected over 12 years by a public ringing scheme according to the protocol of the South African Ringing Institute (SAFRING) (de Beer et al. 2001). The data encompass 16 major vegetation units within nine bioregions within four biomes (Table 2) located from 21°S to 34°S. We hypothesized that survival would be lower in the north of the latitudinal range than in the south, and that the timing of rainfall, the seasonality of the environment, and the migratory strategy of the different populations would influence survival. We predicted that survival would be higher in the area with austral summer rainfall than in the area with winter rainfall and lowest in the areas with irregular rainfall, where the populations are sedentary (Dean 2005). The breeding season of the African reed warbler starts from August onwards, that is, after the austral winter, when the migratory populations return (Dean 2005). Winter rainfall might provide better breeding habitat, that is, denser, higher, and greener reed beds (Eising et al. 2001), and a good food supply early in the season, but summer rainfall would provide a longer period of adequate food supply for adults, which would also leave migrants fitter for migration (Newton 2006). We expected survival to be highest in the least seasonal environments and higher in migratory than in sedentary populations. Although migration is hazardous (Dobson 1990; Newton 2004, 2006; Leisler and Schultze-Hagen 2011), these species tend to have shorter breeding seasons and produce fewer young and sedentary species must endure deteriorating conditions (Alerstam and Högstedt 1982).

**Table 2:** Climate details of the bioregions/vegetation units of the capture sites of the African reed warbler and estimated mean survival during 1998–2010.

Biome	Bioregion	Vegetation unit	Sites (Fig. 1)	Timing P	MAP (mm)	APCV (%)	MAT (°C)	MAFD (days)	Seasonality “score”^1^	Φ	95% CRI
Desert	^*^Central–western Plains^2^		2	Irregular	<50	>100	17.0	0	+	0.69	0.45–0.89
Grassland	Mesic Highveld	Egoli Granite	4	Summer	682	26	16.0	29	±	0.70	0.51–0.87
		Rand Highveld	6	Summer	654	27	15.8	28	±	0.72	0.50–0.89
		Soweto Highveld	7	Summer	662	27	14.8	41	+	0.53	0.34–0.75
		Wakkerstroom Montane	8	Summer	902	22	14.1	31	±	0.65	0.45–0.85
		Eastern Free State Sandy	11	Summer	701	26	13.6	51	+	0.49	0.18–0.80
	Dry Highveld	Carletonville Dolomite	5	Summer	593	28	16.1	37	+	0.83	0.62–0.97
		Winburg Grassy Shrubland	12	Summer	495	31	15.3	41	+	0.71	0.51–0.88
	Sub-escarpment	KZN Highland Thornveld	9	Summer	752	25	16.5	15	−	0.80	0.59–0.95
		Northern KZN Moist	10	Summer	836	23	16.2	20	−	0.59	0.34–0.82
Savanna	# Arid woodland		1	Summer	250–650	?	?	?	?^3^	0.57	0.35–0.79
	Sub-escarpment	Ngongoni Veld	13	Summer	888	22	17.7	2	−	0.76	0.58–0.91
	Central Bushveld	Dwaalboom Thornveld	3	Summer	551	29	19.4	19	±	0.49	0.25–0.75
Fynbos	Southwest Fynbos	Swartland Alluvium	15	Winter	656	27	17.1	3	−	0.65	0.34–0.90
	WC Renosterveld	Swartland Shale	14	Winter	430	32	17.2	3	−	0.57	0.36–0.79
		Swartland Granite	16	Winter	520	30	16.3	3	−	0.75	0.57–0.89

P, precipitation; MAP, mean annual precipitation; APCV, annual variation precipitation coefficient; MAT, mean annual temperature; MAFD, mean annual frost days; Φ, mean survival; CRI, credible Interval; KZN, KwaZulu-Natal; WC, west coast; +, high; ±, intermediate; -, low; ?, not available.

1 Seasonality was “scored” by adding up APCV, MAT, and MAFD (range 41.9–117.0). These values were binned into low (41.9–50), intermediate (50–80), and high (80–117).

2 Fog (visibility ≤1000 m) 100–125 days per year. This could indicate a less seasonal environment than expected based on the seasonality “score”.

3 Because the climatic details were not available, this site was omitted from Fig. 3.

References: ^*^Mendelsohn et al. 2003; #Allan et al. 1997; Mucina and Rutherford 2006.

## Materials and Methods

### Data

**Figure 1:**
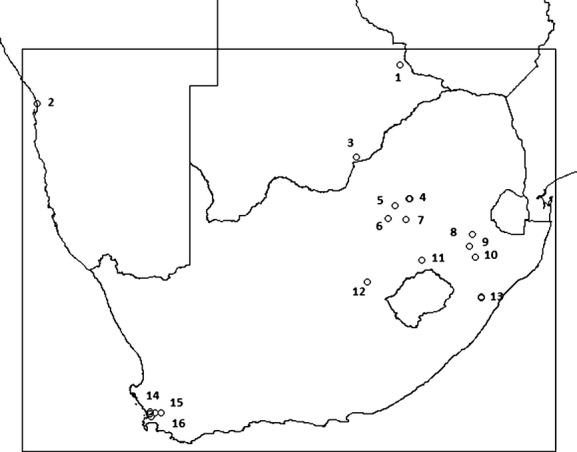
Mist-netting locations analysed in this study of the African reed warbler in southern Africa (1998–2010). The numbers indicate capture sites pooled by proximity within one vegetation unit. Sites 1, 4, and 13 are two locations each less than 0.17 decimal degrees apart; site 14 contains three locations less than 0.17 degrees apart.

From August 1998 to July 2010, 9921 individual adult African reed warblers (11,598 captures) were caught in mist nets throughout the year in southern Africa (12 capture occasions August–July). These captures were made by licensed ringers according to the SAFRING protocol but not within a designed geographical scheme. We examined capture effort at each location to avoid bias in the survival estimates through incidental mist netting. Capture effort ranged from 1 day to 120 days during the entire study period. Twenty-one locations were selected where capture effort was 24 days or more from 1998 to 2010 (circles in Fig. 1). Recaptures confirmed earlier observed high breeding site fidelity (Eising et al. 2001). Except for five individuals, recaptures of the same individual between occasions were within a radius of 0.17 decimal degrees (10 min South and East) of the original capture. We, therefore, included captures made within this radius of 0.17 decimal degrees of the 21 high effort locations. The subsequent dataset comprised of 6951 individual adult reed warblers (7,816 captures), of which 701 individuals were recaptured at least once in subsequent occasions. Table S1 lists captures per site per year.

The biomes/bioregions/vegetation units of the locations in South Africa, and one in Botswana by proximity, were extracted with ArcView GIS 3.1 (ESRI 1999) from the latest vegetation map (Mucina and Rutherford 2006); for Namibia the map in Mendelsohn et al. 2003 and for Botswana the map in Allan et al. 1997 were used (Table 2). When locations were within 0.17 decimal degrees of others and all within the same vegetation unit, they were viewed as one site (numbers in Fig. 1). Because no direct measurement of seasonality was available, we used the climate details of the vegetation types and “scored” seasonality by adding up annual precipitation coefficient of variation (APCV), mean annual temperature (MAT), and mean annual frost days (MAFD) (Table 2).

Atlas data indicate that African reed warbler populations are migratory in the south of southern Africa, roughly below 26°S (Harrison 1997; Dean 2005). We used this latitude to separate migratory populations from nonmigratory populations. This split locations in Namibia and Botswana (sites 1–3, Fig. 1) from locations in South Africa, resulting in 1,021 nonmigratory captures versus 6,795 migratory captures.

### Data analysis

We used capture–mark–recapture (CMR) models for open populations to estimate apparent adult survival probability (hereafter survival), “apparent” because mortality and permanent immigration are confounded. The models assume individual homogeneity in survival and recapture and no lost or missed marks, and condition on first capture (Lebreton et al. 1992). We first pooled the data from all sites and assessed goodness-of-fit (GOF) of the global Cormack–Jolly–Seber model (i.e., fully time-dependent survival *ϕ*_*t*_ and recapture p_*t*_ (*ϕ*_*t*_p_*t*_)) with Program U-CARE 2.3.2 (Choquet et al. 2009). The directional *z*-test for transience (3.SR) was significant (*P *=* *0). Transients, as opposed to residents, are individuals with a zero survival probability after initial capture (Pradel et al. 1997). By necessity, mist nets are placed along the edge of reed beds. In large patches of suitable habitat capture at the edge, where a territorial bird like the African reed warbler that nests in close proximity to conspecifics (Urban et al. 1997; Eising et al. 2001) only occasionally forages, might result in low recapture while placement in the middle of breeding territories (e.g., fragmented reed beds) would net much higher numbers, leading to an excess of individuals that are only captured once (Buckland and Baillie 1987). Passerine mist-netting data often show transience and not accounting for it will lead to underestimation of survival (Buckland and Baillie 1987). With the removal of 3.SR, the overall GOF no longer showed lack of fit (*P *=* *0.23).

We used multistate capture–recapture models (Gimenez et al. 2007) to account for transience and to investigate our hypotheses (sites grouped according to rainfall timing, vegetation units and migratory strategy). We considered a three-state model where the state transition matrix is given by

**Table 3:** 

	True state (*t* + 1)		
True state (*t*)	Initial	Resident	Dead
Initial	0	*ϕ* * *ψ*	1−(*ϕ* * *ψ*)
Resident	0	*ϕ*	1−*ϕ*
Dead	0	0	1

(*ϕ* – survival, *ψ* – transition probability) and the observation matrix by

**Table 4:** 

	Observed state (*t* + 1)		
True state (*t*)	Seen as *I*	Seen as *R*	Not seen
Initial (*I*)	0	0	1
Resident (*R*)	0	p	1-p
Dead	0	0	1

(p– recapture probability). We then developed a hierarchical model with additive random site and year effects to quantify the spatial and temporal variation in survival (the sparse data prohibited the use of an interactive model):


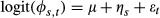


where *ϕ*_*s,t*_ is the survival probability from time *t* to *t + 1* in site *s*; *μ* is the overall mean survival on the logit scale. *η*_*s*_ and *ε*_*t*_ are the site and year random effects, respectively, that are independently normally distributed (i.e., *η*_*s*_ ∼ *N*(0, 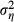), and *ε*_*t*_ ∼ *N*(0, 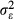)). 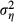 and 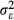 are the spatial and temporal variations (on the logit scale) in survival. Testing our hypotheses required estimating mean survival across groups of sites with similar rainfall regime or migratory status. We calculated these survival rates from the posterior distributions of the site-specific survival rates.

We treated the residence probability (*ψ*) constant over time, but allowed it to differ among sites. Because the data were sparse, we used a single random time effect to model spatio-temporal variation in recapture probability at all sites. That is,


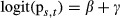


where p_*s,t*_ is the recapture probability at time *t* in site *s*, *β* is the overall mean recapture on the logit scale. *γ* is the spatio-temporal random effect that is independently normally distributed (i.e., *γ* ∼ *N*(0, 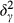 ), and 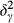 is the spatio-temporal variation in recapture probability (on the logit scale). Residence and recapture probability were considered nuisance parameters.

We implemented the models in a Bayesian framework (King et al. 2010; Kéry and Schaub 2012), assuming noninformative priors for all parameters. We specified uniform priors (U[-5,5]) for the overall mean logit survival and recapture probabilities, a uniform prior (U[0,1]) for the residence probability, and uniform priors (U[0,5]) for the standard deviation parameters (see Appendix S1 for details). We ran three independent chains of length 100,000 with a burn-in of 50,000 and a thinning rate of 20. The Brooks–Gelman–Rubin diagnostic statistic (Brooks and Gelman 1998) and the diagnostic plots (trace plots, density plots, and autocorrelation plots) showed no lack of convergence. All the analyses were performed in JAGS 3.3.0 (Plummer 2003) via R package R2jags (Su and Yajima 2012). The R and JAGS code used are available in Appendix S1.

## Results

**Figure 2:**
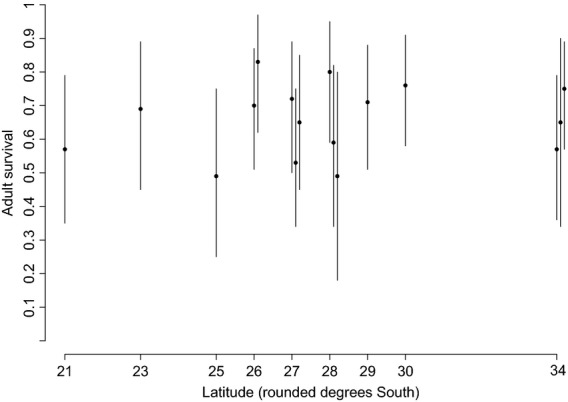
Adult survival of the African reed warbler at capture sites across southern Africa (mean 1998–2010). Sites at the same rounded latitude were separated to show the 95% credible intervals (vertical lines).

**Figure 3:**
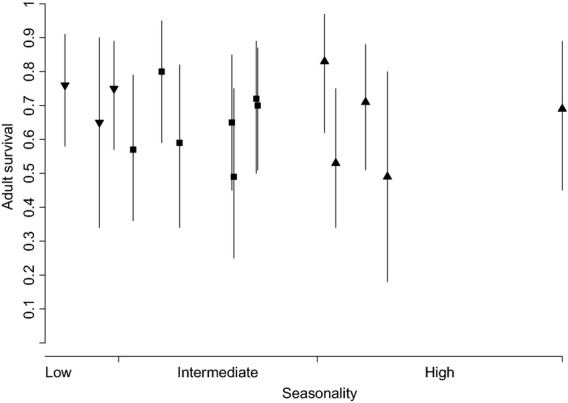
Adult survival of the African reed warbler in environments with varying seasonality (mean survival 1998–2010; 95% credible interval as vertical lines). Site 1 in arid woodland was omitted due to unavailable climatic data. Seasonality was “scored” by adding up annual precipitation variation, mean temperature, and frost days (see Table 2 for details).

Mean adult survival of the African reed warbler was estimated at 0.67 (95% credible interval (CRI): 0.47–0.85). The estimated spatial and temporal standard deviations of the logit survival were 0.70 (95% CRI: 0.33–1.27) and 1.08 (95% CRI: 0.52–2.35), respectively. Variation in survival was unrelated to latitude (Fig. 2). Survival of populations at the same latitude (rounded to degrees) differed widely, for instance from 0.49 (95% CRI: 0.18–0.80) to 0.80 (95% CRI: 0.59–0.95) at 28°S (Fig. 2). Estimated survival per vegetation unit differed considerably with a minimum of 0.49 (95% CRI: 0.18–0.80) in Eastern Free State Sandy Grassland and Dwaalboom Thornveld and a maximum of 0.83 (95% CRI: 0.62–0.97) in Carletonville Dolomite Grassland, but there was no relationship between survival and seasonality (Table 2; Fig. 3). Survival did not differ significantly, or widely, between rainfall regimes: 0.66 (95% CRI: 0.46–0.83) in the winter rainfall (peak May–August) areas (sites 14–16, Fig. 1, Table 2), 0.65 (95% CRI: 0.50–0.82) in the summer rainfall areas (sites 1, 3–13), and 0.69 (95% CRI: 0.51–0.87) in the irregular rainfall area (site 2). On average, migratory populations (sites 4–16) tended to survive better than sedentary populations (sites 1–3), but the difference was not significant as reflected by the overlap of the 95% credible intervals (migratory: 0.67 (95% CRI: 0.52–0.84); sedentary: 0.59 (95% CRI: 0.39–0.78)).

The estimated residence probability varied from a range of 0.25 (95% CRI: 0.04–0.74) for site 15 to 0.86 (95% CRI: 0.65–0.99) for site 4. Because of the sparseness of the data, most residence probability estimates, and a few survival estimates, showed low precision (wide CRIs). The estimated mean recapture probability was 0.12 (95% CRI: 0.08–0.15) with a spatio-temporal standard deviation on the logit scale of 1.21 (95% CRI: 0.97–1.50).

## Discussion

The African subcontinent lends itself well to comparative studies of avian life histories as it covers nine terrestrial biomes with a wide range of climate regimes (Mucina and Rutherford 2006). As a habitat specialist of azonal wetlands, the African reed warbler was a suitable candidate to investigate the influence of environmental seasonality on life-history traits. We found substantial variation in survival among 16 vegetation units within four biomes located between 21°S and 34°S ranging from 0.49 to 0.83 (Table 2). Our findings (Fig. 2) did not reflect the global interspecific pattern of higher survival toward lower latitudes as found, for instance, by Peach et al. (2001) for African and European insectivores and Stevens et al. (2013) for Afrotropical and similar-sized temperate species. The latitudinal band in our study may have been too narrow to detect such a latitudinal gradient, but there was no indication of a trend in the expected direction. Moreau (1944) compared published clutch sizes between the equator and South Africa and concluded that there was evidence for a real but slight latitudinal gradient. Saracco et al. (2012) found spatial variation in survival across the North American continent for the common yellowthroat *Geothlypis trichas* – another partially migratory and similar-sized warbler found mainly in reed beds (Dunn and Alderfer 2008; Sinclair and Ryan 2009). Although the ranges of estimated survival rates for yellowthroats in North America and reed warblers in southern Africa overlap (0.35–0.61 vs. 0.49–0.83), the extremes do conform to the general trends of higher survival of southern hemisphere species compared with northern, temperate species (Brawn et al. 1999; Francis et al. 1999).

Our study did highlight variation in survival at the landscape scale like studies by Ricklefs and Shea (2007), Blake and Loiselle (2008), and Saracco et al. (2012). As the latitudinal seasonality (lower in the South, little in the Tropics and high in the northern, temperate zone) underpins the main hypotheses of the latitudinal gradient in life-history strategies (Moreau 1944; Lack 1947; Skutch 1949; Ashmole 1963), we predicted lower survival in less seasonal environments. We found no such pattern in the substantially differing environments of the African reed warbler populations (Table 2; Fig. 3) or between the different rainfall regimes (Table 2).

There are many potentially interacting influences on life-history traits (Martin 2004; McNamara et al. 2008). Saracco et al. (2012) suggested that part of the spatial variation in survival may be due to life-history differences between sedentary and migratory populations. They found a trend similar to the one we found for the African reed warbler: higher survival among migratory populations than among sedentary populations. By leaving for the wintering grounds when conditions get tough, migratory populations might increase their survival chances to such an extent that it compensates for the added mortality of migration, whereas sedentary populations are exposed to prevailing conditions in situ all year round (Alerstam 1993). Another explanation could be that these birds invest relatively more in reproduction at the expense of survival (Martin 1996; Ricklefs 2000). Thaxter et al. (2006) proposed availability of suitable dispersal habitat as a reason for the spatial differences they found in male Eurasian reed warbler survival in England. Dispersing males would lower resident apparent survival estimates. In the divergent vegetation units across southern Africa, proximity of dispersal habitat could be as different as the environments of the study populations. Eising et al. (2001) observed cooperative breeding with unrelated helpers in the ordinarily monogamous African reed warbler in saturated habitat in an environment with little dispersal opportunity. Less-strenuous breeding could increase annual survival of the breeding pair, and breeding in a group could be safer, for everybody, than breeding alone (Riehl 2013). Another factor – linked to reed beds as breeding habitat – found to influence adult survival is brood parasitism, due to the cost of defense against the parasite and the higher costs of raising the parasite's chick (Leisler and Schultze-Hagen 2011). Stokke et al. (2007); found a positive relationship between host density and the parasitism rate among 16 Eurasian reed warbler populations in Europe. Parasitism risk increased with decreasing distance of tree-top perches – from which the female cuckoo surveys potential victims – to Eurasian reed warbler breeding populations (Welbergen and Davies 2009). More extensive and “pure” reed beds were the least parasitized (Leisler and Schultze-Hagen 2011). Brood parasitism in African reed warbler nests by Klaas's Cuckoos *Chrysococcyx klaas* has been observed in East Africa (Urban et al. 1997).

This study revealed large spatial variation in survival in divergent environments across the African subcontinent and an indication of variation due to migratory strategy. Even with sparse data (average recapture similar to its sister taxon – Buckland and Baillie 1987), our hierarchical modeling approach was able to estimate spatial variation with fairly high precision. We did not find a clear pattern of higher survival in less seasonal environments. As seasonality can only be regarded as a proxy for food supply, quantitative data on this combined with additional data on, for example, dispersal and brood parasitism potential in the vegetation units could aid understanding of the ecological constraints that influence a life-history trait that is a major driver of population dynamics (Baillie and Schaub 2009). Incorporating recording of distance to the nearest dispersal habitat and tree presence within the reed beds into the existing protocol of CMR data collection would be relatively simple. In this manner, a geographically uncoordinated public ringing scheme across a vast subcontinent – where resources for detailed field studies are scarce – could extract important information, as our study already showed, and contribute to the study of life-history strategy and its evolution from a relatively poorly studied region (Martin 1996).

## Supporting Information

Appendix S1 The R and JAGS code used for the African reed warbler survival analysis.

Table S1 Captures per site per occasion of the African reed warbler (1998–2010) in southern Africa.
