# Supplementary material for: Does seasonality drive spatial patterns in demography? Variation in survival in African reed warblers Acrocephalus baeticatus across southern Africa does not reflect global patterns
Source: Ecol Evol. 2014 Feb 23;4(7):889–98. doi: 10.1002/ece3.958 (PMC3997307; doi:10.1002/ece3.958)
Supplement: Appendix S1 — The R and JAGS code used for the African reed warbler survival analysis. [file ece30004-0889-sd1.docx]

**Appendix S1.** The R and JAGS code used for the African reed warbler survival analysis

#**********************************************************************************

# Hierarchical capture-recapture model

# Additive random site and year effects to study the spatial and temporal

# variation in survival

# Residence probability assumed to be constant over time, but allowed to

# differ among sites

# Random temporal variation (assumed to be the same in all sites) in the recapture

# probability

#**********************************************************************************

#********************************************

# Specify model in BUGS language

#*******************************************

model {

#----------------------------------------

# Parameters:

# psi.r: probability of an individual being a resident

# phi.g: resident survival probability per group

# p.g: group recapture probability

#----------------------------------------

# States (S):

# 1 initial state (transients and residents)

# 2 alive as resident

# 3 dead

# Observations (O):

# 1 seen as initial state

# 2 seen as resident

# 3 not seen

#----------------------------------------

#*****************************

# Priors and constraints

#*****************************

for (i in 1:nind){

for (t in 1:(n.occasions-1)){

logit(phi.r[i,t]) <- lphi[group[i],t] # survival

psi.r[i,t] <- psi.g[group[i]] # residence probability

logit(p[i,t]) <- lp[group[i],t] # recapture

} #t

} #i

for (u in 1:g){

for (t in 1:(n.occasions-1)){

lphi[u,t] <- lmu.phi + beta.phi[u] + eps.phi[t]

lp[u,t] <- lmu.p + eps.p[u,t]

eps.p[u,t] ~ dnorm(0,tau.p)T(-5,5)

}#t

beta.phi[u] ~ dnorm(0, tau.phig)T(-5,5)

} # u

for (t in 1:(n.occasions-1)){

eps.phi[t] ~ dnorm(0,tau.phit)T(-5,5)

}#t

# overall mean survival, spatial variation, and temporal variation

lmu.phi ~ dunif(-5,5) ; mu.phi <- exp(lmu.phi)/(1 + exp(lmu.phi))

sig.phig ~ dunif(0,5) ; tau.phig <- pow(sig.phig, -2)

sig.phit ~ dunif(0,5) ; tau.phit <- pow(sig.phit, -2)

# overall mean recapture probability and variation

lmu.p ~ dunif(-5,5) ; mu.p <- exp(lmu.p)/(1 + exp(lmu.p))

sig.p ~ dunif(0,5) ; tau.p <- pow(sig.p, -2)

# residence probability

for (u in 1:g){

psi.g[u] ~ dunif(0,1)

}#u

# derived parameters (site-specific survival)

for (u in 1:g){

mu.phig[u] <- exp(lmu.phi + beta.phi[u])/(1 + exp(lmu.phi + beta.phi[u]))

} # u

#*****************************************************

# Define state-transition and observation matrices

#*****************************************************

for (i in 1:nind){

# Define probabilities of state S(t+1) given S(t)

for (t in 1:(n.occasions-1)){ # loop over time

ps[1,i,t,1] <- 0

ps[1,i,t,2] <- phi.r[i,t] * psi.r[i,t]

ps[1,i,t,3] <- 1-(phi.r[i,t] * psi.r[i,t])

ps[2,i,t,1] <- 0

ps[2,i,t,2] <- phi.r[i,t]

ps[2,i,t,3] <- 1-phi.r[i,t]

ps[3,i,t,1] <- 0

ps[3,i,t,2] <- 0

ps[3,i,t,3] <- 1

# Define probabilities of O(t) given S(t)

po[1,i,t,1] <- 0

po[1,i,t,2] <- 0

po[1,i,t,3] <- 1

po[2,i,t,1] <- 0

po[2,i,t,2] <- p[i,t]

po[2,i,t,3] <- 1-p[i,t]

po[3,i,t,1] <- 0

po[3,i,t,2] <- 0

po[3,i,t,3] <- 1

} #t

} #i

# State-space model likelihood

for (i in 1:nind){

z[i,f[i]] <- Y[i,f[i]]

for (t in (f[i]+1):n.occasions){ # loop over time

# State equation: draw S(t) given S(t-1)

z[i,t] ~ dcat(ps[z[i,t-1],i, t-1,])

# Observation equation: draw O(t) given S(t)

Y[i,t] ~ dcat(po[z[i,t],i, t-1,])

} #t

} #i

}# model

#********************************END *************************************
