# Supplementary material for: Does seasonality drive spatial patterns in demography? Variation in survival in African reed warblers Acrocephalus baeticatus across southern Africa does not reflect global patterns
Source: Ecol Evol. 2014 Feb 23;4(7):889–98. doi: 10.1002/ece3.958 (PMC3997307; doi:10.1002/ece3.958)
Supplement: Table S1 — Captures per site per occasion of the African reed warbler (1998–2010) in southern Africa. [file ece30004-0889-sd2.docx]

**Table S2.** Captures per site per occasion of the African reed warbler (1998-2010) in southern Africa (initial captures in the last occasion were omitted).

| Occasion | 1 | 2 | 3 | 4 | 5 | 6 | 7 | 8 | 9 | 10 | 11 | 12 |
| --- | --- | --- | --- | --- | --- | --- | --- | --- | --- | --- | --- | --- |
| Site |  |  |  |  |  |  |  |  |  |  |  |  |
| 1 | 58 | 51 | 114 | 121 | 63 | 9 | 4 | 4 | 0 | 0 | 1 | 0 |
| 2 | 2 | 6 | 0 | 3 | 25 | 60 | 38 | 50 | 26 | 23 | 0 | 1 |
| 3 | 140 | 39 | 151 | 0 | 21 | 0 | 0 | 0 | 0 | 0 | 0 | 0 |
| 4 | 17 | 32 | 47 | 34 | 43 | 49 | 31 | 37 | 26 | 17 | 32 | 11 |
| 5 | 0 | 0 | 0 | 0 | 1 | 7 | 13 | 10 | 30 | 23 | 10 | 5 |
| 6 | 43 | 13 | 0 | 7 | 22 | 211 | 158 | 82 | 33 | 33 | 114 | 2 |
| 7 | 50 | 67 | 16 | 0 | 6 | 313 | 167 | 62 | 78 | 30 | 41 | 7 |
| 8 | 0 | 7 | 82 | 157 | 135 | 216 | 206 | 41 | 0 | 0 | 53 | 1 |
| 9 | 0 | 0 | 0 | 0 | 0 | 8 | 34 | 43 | 43 | 72 | 9 | 2 |
| 10 | 0 | 0 | 12 | 6 | 94 | 43 | 69 | 0 | 0 | 0 | 10 | 1 |
| 11 | 0 | 21 | 0 | 3 | 20 | 35 | 83 | 23 | 17 | 6 | 4 | 0 |
| 12 | 42 | 66 | 43 | 38 | 9 | 34 | 19 | 21 | 7 | 5 | 10 | 0 |
| 13 | 60 | 104 | 38 | 70 | 135 | 32 | 39 | 101 | 86 | 63 | 84 | 6 |
| 14 | 95 | 70 | 77 | 78 | 133 | 74 | 84 | 42 | 25 | 15 | 13 | 0 |
| 15 | 10 | 0 | 5 | 13 | 13 | 6 | 0 | 4 | 3 | 10 | 0 | 0 |
| 16 | 89 | 127 | 73 | 84 | 70 | 122 | 88 | 100 | 85 | 84 | 77 | 0 |
